# Telomere biology disorders

**DOI:** 10.1038/s41525-021-00198-5

**Published:** 2021-05-28

**Authors:** Michelle L. W. Kam, Trang T. T. Nguyen, Joanne Y. Y. Ngeow

**Affiliations:** 1 Department of Respiratory and Critical Care Medicine, Singapore General Hospital, Singapore, Singapore; 2 Lee Kong Chian School of Medicine, Nanyang Technological University, Singapore, Singapore; 3 Cancer Genetics Service, Division of Medical Oncology, National Cancer Centre, Singapore, Singapore

**Keywords:** Disease genetics, Respiratory tract diseases, Anaemia, Medical genetics

## Abstract

Telomere biology disorders (TBD) are a heterogeneous group of diseases arising from germline mutations affecting genes involved in telomere maintenance. Telomeres are DNA-protein structures at chromosome ends that maintain chromosome stability; their length affects cell replicative potential and senescence. A constellation of bone marrow failure, pulmonary fibrosis, liver cirrhosis and premature greying is suggestive, however incomplete penetrance results in highly variable manifestations, with idiopathic pulmonary fibrosis as the most common presentation. Currently, the true extent of TBD burden is unknown as there is no established diagnostic criteria and the disorder often is unrecognised and underdiagnosed. There is no gold standard for measuring telomere length and not all TBD-related mutations have been identified. There is no specific cure and the only treatment is organ transplantation, which has poor outcomes. This review summarises the current literature and discusses gaps in understanding and areas of need in managing TBD.

## Introduction

Telomeres are noncoding repeats of the *TTAGGG* nucleotide sequence at chromosomal ends that provide genomic stability^1,2^. They shorten with each cellular division, triggering senescence and apoptosis when critically short^1,3,4^. Genetic mutations affecting telomere homoeostasis, thus, result in disorders of premature aging, with various manifestations like bone marrow failure, pulmonary fibrosis, liver cirrhosis, premature greying and increased cancer risk^5–9^. Various terms such as short telomere syndrome, telomeropathies and telomere biology disorders (TBD) have been employed to describe these diseases; in this review, we use the term TBD as it reflects the spectrum of the disorders and pathophysiology^4,8^.

The archetypal TBD is dyskeratosis congenita (DC), which was first described by Zinsser in the early twentieth century as a mucocutaneous triad of skin pigmentation, nail dystrophy and oral leukoplakia, with other systemic features identified later^8^. DC accounts for less than 5% of all TBD and has an estimated incidence of 1 in 1,000,000^8^. It is estimated that 41% of familial mixed haematologic and interstitial lung disease (ILD) and 3% of familial haematologic disorders are due to TBD, however the protean presentation makes recognition challenging and the overall incidence is unknown^6^.

**Fig. 1:**
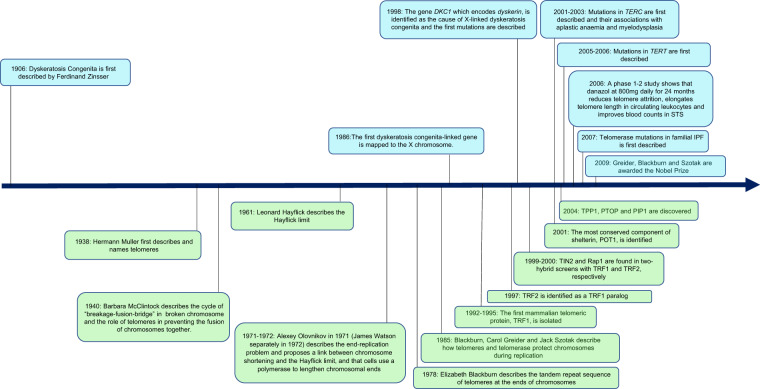
Timeline of significant discoveries and developments in telomere biology and their clinical relevance. Since dyskeratosis congenita was first described in the early 1900s, there have been significant developments in the knowledge of telomere biology over the last century. It is now known that telomeres are DNA-protein structures found at the ends of chromosomes and provide stability to chromosomes and prevent deterioration during cellular replication. Telomere length attrition thus leads to cellular senescence and triggers cell death pathways. Telomere biology disorders are a group of monogenic disorders of premature aging arising due to accelerated shortening of telomere lengths. It has a highly variable presentation due to various germline mutations and incomplete penetrance. Clinical phenotypes vary from multisystem disorders presenting in childhood such as dyskeratosis congenita and single organ disorders such as idiopathic pulmonary fibrosis, which may present in late adulthood. Despite the significant advancement, the true extent of the syndrome remains unknown and further genetic mutations have yet to be identified.

The first DC-related mutations were described when *DKC1* mutations were identified as the cause of X-linked DC^10^. It is now known that DC and other TBD arise from germline mutations affecting telomeres^5–7,10–19^. Inheritance can be X-linked recessive, autosomal dominant (AD) or autosomal recessive (AR) and incomplete penetrance results in manifestations of varying degrees of severity at different time points within the same pedigree^5,8,17,20^. Anticipation is also demonstrated with earlier, more severe presentations due to progressive telomere shortening through the generations^5,20^. Although there is now a greater understanding of telomere biology (Fig. 1), many mechanisms of telomere dysfunction have not been identified. This review summarises the current literature and discusses gaps in understanding and areas of need in managing TBD.

## Mechanisms/pathophysiology

### Telomere biology

Telomeres are crucial for chromosomal stability and protect them from deterioration during mitosis or aberrant fusion with neighbouring chromosomes^1,4^. They address the “end-replication” problem that describes DNA loss and progressive chromosomal shortening with each cell division^3^. Linear DNA and broken chromosomes are unstable and have a propensity to fuse^21^. Critical telomere shortening or broken chromosomes, thus, result in aberrant recombination with end-to-end fusions and “breakage-fusion-bridge” cycles, which are termed “crisis”^1,4,21^. Crisis is characterised by replicative senescence, chromosome end-to-end fusions and extensive apoptosis, causing uneven derivative chromosomes and genomic instability^21^.

**Fig. 2:**
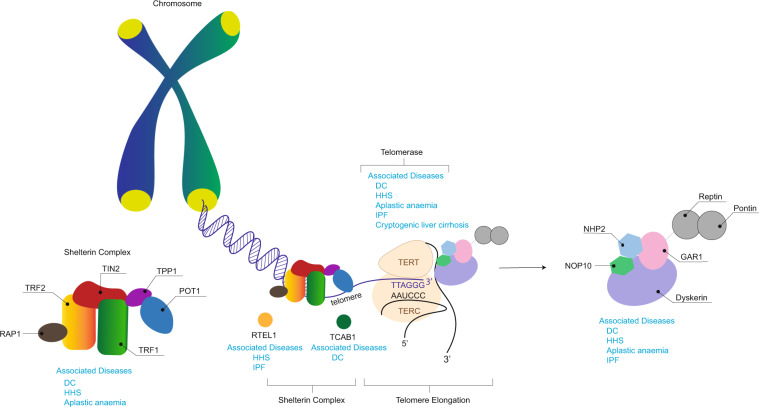
Telomere and telomerase complex components and their associated diseases. Telomeres are noncoding tandem repeats of the sequence *TTAGGG*, found at the ends of chromosomes in a duplex “D-loop-T-loop” configuration. RTEL1 is a helicase that disrupts T-loops for telomere replication and repair. Shelterin protects telomeres from DNA damage surveillance and comprises of six polypeptide components: TRF1, TRF2, RAP1, TIN2, TPP1 and POT1. Telomerase comprises of the essential components TERT and TERC, which synthesise telomeres and maintain telomere length. Dyskerin forms a complex with NHP2, NOP10 and GAR1. It binds to TERC to stabilise the telomerase complex. TCAB1 regulates the recruitment of telomerase to telomeres. Mutations in any of these components results telomere dysfunction, manifesting as telomere biology disorders. DC, dyskeratosis congenita; GAR1, H/ACA ribonucleoprotein complex subunit 1; HHS, Hoyeraal–Hreidarsson Syndrome; IPF, idiopathic pulmonary fibrosis; NHP2, H/ACA ribonucleoprotein complex subunit 2; NOP10, H/ACA ribonucleoprotein complex subunit 3; POT1, protection of telomeres 1; RAP1, repressor/activator protein 1; RTEL1, regulator of telomere length 1; TCAB1, Telomerase Cajal body protein 1; TERT, telomerase reverse transcriptase; TERC, telomerase RNA component; TIN2, TRF1-interacting nuclear protein 2; TPP1, TIN2-interacting protein 1; TRF1, telomere repeat-binding factor 1; TRF2, telomere repeat-binding factor 2.

The telomere structure may result in DNA damage responses recognising their ends as double-stranded DNA breaks and various mechanisms help to maintain its integrity (Fig. 2). Telomeres have a stabilising “D-loop-T-loop” configuration; the T-loop is formed by insertion of the single-stranded G-strand terminus into the double-stranded telomere sequence, it displaces the sequence strand of the duplex telomeric DNA and forms the D-loop at the point of insertion^22^. Telomeres are protected by shelterin, which is a protein complex that generates and stabilises the “T-loop” structure, and facilitates and regulates telomere elongation^4,23^. It comprises six subunits, namely, telomere repeat-binding factor 1 (TRF1), telomere repeat-binding factor 2 (TRF2), repressor/activator protein 1 (RAP1), TRF1-interacting nuclear protein 2 (TIN2), TIN2-interacting protein 1 (TPP1) and protection of telomeres 1 (POT1)^23^. TRF1, TRF2 and POT1 localise the telomeric sequence, while TIN2, TPP1 and RAP1 form a complex that distinguishes telomeres from sites of DNA damage^4,23^. Ku is a DNA-end-binding heterodimer involved in DNA repair, but also associates with telomeres and protects them from recombination and degradation, regulates telomere addition and maintains telomere length (TL)^24^.

Telomerase is a ribonucleoprotein complex that adds telomere repeats to chromosomal ends and maintains TL^2^. Its essential components are telomerase reverse transcriptase (TERT) and an RNA component (TERC), which provides the template for nucleotide addition^1^. Immature TERC is non-functional with short extended oligoadenylated forms^25^. Its maturation involves PARN, which removes oligoadenylated tails that mark nuclear RNAs for exosome-dependent degradation^25^. In contrast, PAPD5 oligoadenylates immature TERC and thus targets it for exosome degradation^26^. The nuclear exome targeting (NEXT) complex directs a subset of noncoding RNAs for exosomal degradation and is also involved in TERC maturation^27^. It comprises three subunits, RNA helicase Mtr4p (hMTR4), RNA recognition motif (RRM)-containing RBM7 and zinc-knuckle ZCCHC8 proteins^27,28^. *PARN* “loss-of-function” mutations and ZCCHC8 loss result in reduced mature TERC, while PAPD5 increases TERC and increases TL^26,28,29^.

Dyskerin, which is encoded by *DKC*, binds the H/ACA box of TERC to stabilise the telomerase complex^4,10,30^. Dyskerin forms a core complex with NHP2, NOP10 and GAR1, and associates with other proteins like NAF1 and TCAB1 for assembly, trafficking, recruitment and stabilisation^4,30–32^. There are other important components of telomere replication such as RTEL1 (regulator of telomere length 1) and CST (CTC1-STN1-TEN1). RTEL1 is a helicase that disrupts the T-loop during telomere replication, and its dysfunction results in stalling of replication and T-loop excision, shortening TL^33^. CST is a single-stranded DNA complex consisting of conserved telomere protection component 1 (CTC1), suppressor of cdc thirteen 1 (STN1) and telomeric pathway with STN1 (TEN1)^34^. CST is essential for synthesising the complementary C-strand and limiting telomerase action to prevent G-strand overhang overextension, thus preventing telomeric DNA damage signalling^34,35^. Germline mutations causing dysregulation of any of the components involved in telomere homoeostasis thus results in TBD (Table 1).

**Table 1:** Summary of genetic mutations described in telomere biology disorders.

Gene mutation	Product	Inheritance	Disease and phenotype	Frequency	Comment
*TERT*^5–7,17,47,50,54,59^	TERT	Autosomal dominant Autosomal recessive	DC^a^ IPF^b^/FIP^c^ AA^d^/MDS^e^ Familial Liver Cirrhosis DC HHS^f^	15–25% 8–15% (familial) 1–3% (sporadic) 3–5% (AA) 20% (familial MDS-AML^i^) Rare, unknown Rare	*TERT* and *TERC* mutations are the most common TBD mutations and present later in life.
*TERC*^5,6,20,47,50,54,59^	Telomerase RNA component	Autosomal dominant	DC IPF/FIP AA/MDS Familial Liver Cirrhosis	10% 8–15% (familial) 1–3% (sporadic) 3–5% (AA) 20% (familial MDS-AML) rare, unknown	*TERT* and *TERC* mutations are the most common TBD mutations and present later in life.
*DKC1*^10,48^	Dyskerin	X-linked recessive	DC HHS IPF/FIP	15–25% rare <1%	Described in severe X-linked DC, which presents in the first decade of life
*RTEL1*^19,29,50^	RTEL1	Autosomal recessive Autosomal dominant	DC HHS IPF/FIP	Rare Rare 5–10%	–
*TINF2*^16,49^	TIN2	Autosomal dominant	DC HHS RS^g^ IPF/FIP	Rare Rare Rare <1%	Results in very short telomeres. Presentation ranges from milder disease (e.g., FIP) to early-onset severe diseases (e.g., HHS, DC). Only mutation identified with RS
*PARN*^11,29^	PARN	Autosomal dominant Autosomal recessive	IPF/FIP DC HHS	5% Rare Rare	Biallelic and monoallelic variants are associated with DC, HHS, and IPF, respectively
*ZCCHC8*^28^	ZCCHC8	Autosomal dominant	FIP	Rare	–
*NAF1*	NAF1	Autosomal dominant	IPF/FIP, CPFE^h^	<1%	–
*NOP10*^13^ *NHP2*^18^ *WRAP53*^32^ *CTC1*^12,35^	NOP10 NHP2 TCAB1 CTC1	Autosomal recessive	DC	Rare	*CTC1* has been described in Coats’ plus
*ACD*^14^	TPP1	Autosomal dominant Autosomal recessive	AA DC HHA	Rare Rare	–

References are in numerical superscript.

^a^Dyskeratosis congenita.

^b^Idiopathic pulmonary fibrosis.

^c^Familial interstitial pneumonia.

^d^Aplastic anaemia.

^e^Myelodysplastic syndrome.

^f^Hoyeraal–Hreidarsson syndrome.

^g^Revesz syndrome.

^h^Combined pulmonary fibrosis and emphysema.

^i^Acute myeloid leukaemia-myelodysplastic syndrome.

### Telomere length influences and effects

The Hayflick limit describes the fixed number of cell replications prior to senescence and apoptosis, which is triggered by short telomeres^1,3^. TL thus reflects somatic cell replicative history and life span, with shorter TL correlating with increasing age^36,37^. At birth, the average leucocyte TL (LTL) is estimated to be 10 kb and attrition rate ranges from 27 to 41 base pairs per year^37,38^. The factors affecting TL and attrition are complex, but genetics has significant influence. Telomeres are longer in individuals with African ancestry and LTL attrition in Down’s syndrome is thrice that of age-matched controls^36,37^. Although TL and variants in non-telomeric regions related to telomere maintenance are hereditable, variable local gene expression across different cell lines results in varying TL within an individual^20,36,37,39^. For example, testis have longer TL than other cell types and higher *TERT* expression, while cells with a higher rate of turnover like blood and lung epithelial cells, have a shorter TL^36^.

**Fig. 3:**
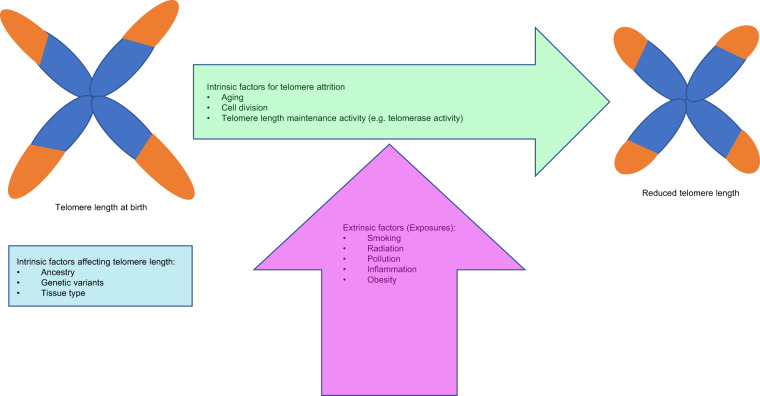
Factors influencing telomere length and attrition. Schema showing how intrinsic factors such as genetic variants influence telomere length and the influence of exposures and intrinsic factors like age, on telomere attrition.

Variation in TL is also demonstrated between twins and family members, suggesting that extrinsic factors also affect TL and attrition^36,39,40^ (Fig. 3). Some of these include obesity, cigarette smoking and environmental exposures^36,38,40^. Exposures and chronic inflammatory states like obesity and recurrent infection, result in reactive oxygen species generation and DNA damage, leading to shorter telomeres and accelerated cellular aging^38,40,41^. Short telomeres are implicated in the degenerative defects of aging and carcinogenesis; however the exact mechanisms have not been fully delineated yet^4,8^.

## Clinical presentation

### Childhood onset syndromes

**Fig. 4:**
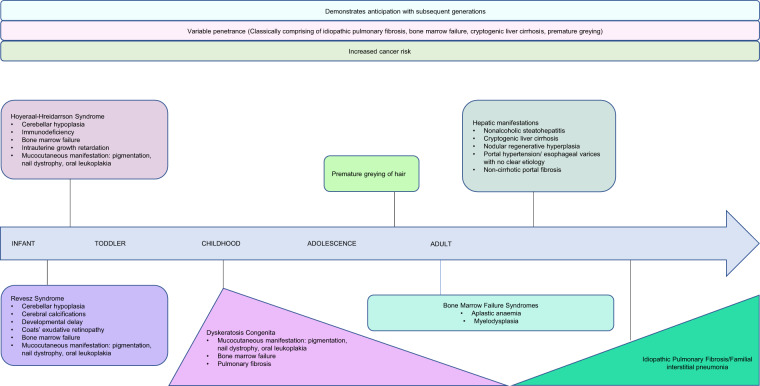
The natural history of telomere biology disorders. Each disorder has its own spectrum of clinical features. Hoyeraal–Hreidarsson Syndrome and Revesz Syndrome present early in childhood and are considered more severe subtypes of dyskeratosis congenita. Dyskeratosis congenita presents commonly in childhood but may manifest at any age. Patients classically present with the mucocutaneous triad of abnormal skin pigmentation, nail dyskeratosis and leukoplakia, and bone marrow failure is the most common cause of death. Idiopathic pulmonary fibrosis is the most common presentation and typically presents after the age of 60. A constellation of premature greying, bone marrow failure, pulmonary fibrosis and cryptogenic liver cirrhosis is suggestive of telomere biology disorders, but due to incomplete penetrance and various inheritance patterns of germline mutations, not all features may be present. The disease demonstrates anticipation with earlier and more severe manifestations down the generations. Across all phenotypes and ages, patients are at an increased risk of cancers.

DC is the best described childhood TBD, it is a syndrome of inherited bone marrow failure with 80–90% developing aplastic anaemia (AA) or bone marrow failure by age 30^8,15–17,20^. Inheritance may be X-linked recessive, AD, or AR, but sporadic cases also occur^8,10,13,17,20^. Although patients classically develop mucocutaneous manifestations and anaemia in adolescence, the severe X-linked form may present within the first decade of life, and presentation may be at any age (Fig. 4)^8,10,13,15–18,20^. There may be multisystem involvement; up to 20% develop pulmonary fibrosis, and other traits include liver disease, premature greying of hair, epiphora, developmental delay, short stature, osteoporosis, avascular necrosis of the hips or shoulders, urethral stenosis and oesophageal stenosis^8,10,13,15–18,20^. Malignancy risk is high and patients are particularly susceptible to epithelial cancers such as head and neck squamous cell carcinoma, anorectal and skin cancers, and haematological malignancies^9,42^. The most common cause of death is bone marrow failure, followed by pulmonary complications and malignancy^8,9,17,20,42^.

The diagnostic criteria for DC are summarised in Table 2^15,16,43^. The severe forms of DC, Hoyeraal–Hreidarrson Syndrome (HHS) and Revesz Syndrome (RS) often present in infancy, and patients often do not survive long enough to develop all the syndromic features or mucocutaneous manifestations^15–17^. HHS presents with cerebellar hypoplasia, immunodeficiency, progressive bone marrow failure and intrauterine growth retardation^8,11,14,15,17,19^. RS has the additional traits of Coats bilateral exudative retinopathy, although cerebral calcifications are often present^8,15,16^. More recently, Coats plus, which overlaps with RS, has been described and is characterised by exudative retinopathy, gastrointestinal vascular ectasia and leukoencephalopathy^12^.

**Table 2:** Summary of dyskeratosis congenita diagnostic criteria.

Diagnosis	Criteria^15,16,43^
DC^a^	1. All three mucocutaneous features: • Abnormal skin pigmentation • Nail dystrophy • Leukoplakia 2. At least 1 out of 3 mucocutaneous feature, combined with bone marrow failure and 2 other somatic features of DC 3. Aplastic anaemia, myelodysplastic syndrome or pulmonary fibrosis associated with a pathogenic telomerase variant 4. 2 or more features seen in DC associated with very short telomeres that are below 1st centile.
Hoyeraal–Hreidarsson Syndrome	Four or more features of: 1. Growth retardation 2. Developmental delay 3. Microcephaly 4. Bone marrow failure 5. Immunodeficiency 6. Cerebellar hypoplasia

^a^Dyskeratosis congenita. References are in numerical superscript.

### Pulmonary manifestations

Idiopathic pulmonary fibrosis (IPF) is the most common TBD manifestation^5,44^. It is a disease of progressive lung fibrosis with a deteriorating disease course and median survival of 3 years^45^. It has a prevalence of 1.25–23.4 per 100,000 in Europe^46^. IPF commonly affects male smokers over 60 and typically has a usual interstitial pneumonia (UIP) pattern on chest high-resolution computed tomography (HRCT) or lung biopsy in the appropriate clinical context^45^.

**Fig. 5:**
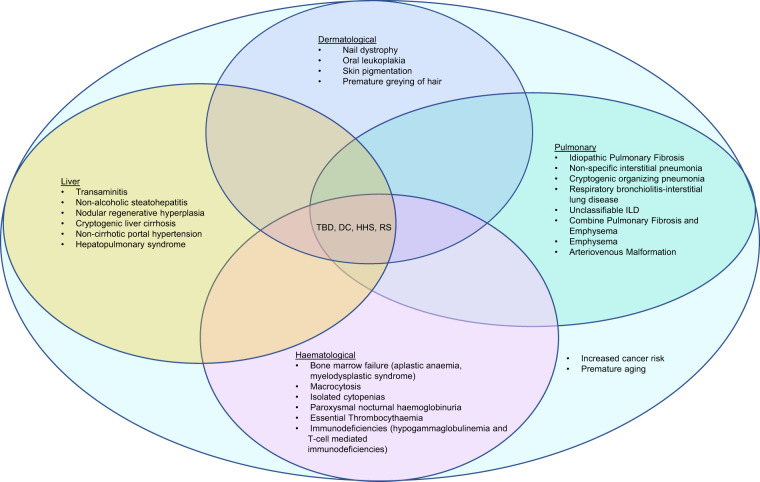
Phenotypes of telomere biology disorders. The heterogenous presentation of telomere biology disorders manifestations may overlap and manifest at various time points or only following certain exposures or insults (e.g., radiation and immunosuppression as preconditioning regimens prior to bone marrow transplant may lead to pulmonary fibrosis in individuals who may have presented with isolated bone marrow failure). Regardless of clinical phenotype, patients are at increased risk of malignancies and disorders of premature aging. DC, dyskeratosis congenita; HHS, Hoyeraal–Hreidarsson Syndrome; ILD, interstitial lung disease; RS, Revesz Syndrome; TBD, telomere biology disorders.

At least 30% of patients with sporadic or familial pulmonary fibrosis have genetic predisposing factors that are known to increase the risk of pulmonary fibrosis^45,47^. *TERT* and *TERC* mutations account for 8–15% of familial and 1–3% of sporadic IPF, while other telomere-related mutations like *NAF1*, *PARN*, *RTEL1*, *DKC1*, *TINF2* and *ZCCHC8* account for 1-3% of familial cases^5,28,29,31,44,47–49^. FIP presents earlier with a more aggressive disease course than sporadic cases, and radiological and histological heterogeneity exists even within a pedigree^5,44^. Non-specific interstitial pneumonia (NSIP), cryptogenic organising pneumonia (COP), respiratory bronchiolitis-interstitial lung disease (RB-ILD), unclassifiable ILD and CPFE have also been described in FIP^5,29,31,44^ (Fig. 5).

TL below the 10th percentile of age-matched controls are found in sporadic and familial IPF with no telomere-related mutations^44^. Other sporadic forms of ILD have also been found to have shorter telomeres and telomere-related mutations^50^. TL below the 10th percentile correlate with poorer outcomes in IPF patients treated with immunosuppression and are associated with faster lung function deterioration, higher mortality and poorer lung transplantation outcomes across the different ILD subtypes^44,50–52^.

More recently, pulmonary arteriovenous malformations (AVM) have been described in patients with DC and TBD even in the absence of hepatopulmonary syndrome (HPS), another known manifestation of TBD^53^.

### Haematological manifestations

Bone marrow failure from AA or myelodysplastic syndrome (MDS) may occur as an isolated manifestation of TBD, most commonly due to *TERT* or *TERC* mutations^7,20,54^. TBD cases who first present with AA are younger than those who first present with IPF, suggesting that AA is a more severe phenotype^5,7,54^. Telomerase-related mutations account for 3–5% of AA and *TERC* mutations have more severe disease; they also have significantly shorter LTL compared to idiopathic and secondary cases of AA^7,54^. A short LTL correlates with poorer outcomes in AA and MDS, including higher rates of transformation to acute leukaemia, poorer survival and higher rates of non-relapse mortality after transplantation^55,56^.

Other haematological conditions described in TBD include macrocytosis, isolated cytopenias, paroxysmal nocturnal haemoglobinuria and essential thrombocythaemia^5,6,8,15,20,54^ (Fig. 5). Immunodeficiencies like hypogammaglobulinemia, lymphopenia and T-cell dysfunction have also been described^8,14–16,57^. It is postulated that underlying immune dysfunction and accelerated immunosenescence increased cancer risk due to failure of cancer surveillance and contribute to poor tolerance of immunosuppression^15,16,52,57^.

### Hepatobiliary manifestations

Up to 40% of patients with TBD have liver involvement^6,58,59^. Manifestations include transaminitis, non-alcoholic steatohepatitis, nodular regenerative hyperplasia, cryptogenic liver cirrhosis, noncirrhotic portal hypertension and HPS (Fig. 5)^6,58–60^. Biopsy findings include inflammatory and fibrotic components, hepatic nodular regeneration, cirrhosis and idiopathic hemosiderosis^58–60^. HPS in TBD typically presents during the first four decades of life, which is earlier than that for pulmonary fibrosis; thus when younger TBD patients present with breathlessness, HPS should be evaluated for, particularly in the absence of pulmonary fibrosis^60^.

Shorter TL have been found in non-alcoholic steatohepatitis, which is postulated to make them vulnerable to a “second hit”^58,59^. Besides telomere-related germline mutations, chronic cell injury and exposures to hepatotoxic drugs and alcohol may accelerate telomere attrition, contributing to progression to liver fibrosis^59^. Similarly, shorter telomeres, *TERC* and *TERT* promoter mutations are linked to the pathogenesis of various hepatobiliary tumours including hepatocellular carcinoma^58,59^.

## Diagnosis

### Measuring telomere length

Most methods that are currently employed measure the average TL^61^. Currently, there is no common reference or gold standard method to measure and trend TL. Furthermore, TL measured using different methods do not correlate well^62^. These factors limit objective comparisons between different cohorts and changes in TL within an individual over time.

The earliest method used to measure TL is terminal restriction fragment (TRF) analysis by Southern blot, which measures the average TRF. TL is calculated by comparing the electrophoresis-separated TRF against known molecular weight markers^63^. This is time consuming and requires substantial amounts of DNA. Furthermore, it overestimates TL as subtelomeric DNA is included in the measurement^61,63^.

Quantitative polymerase chain reaction (qPCR) is most commonly used. It involves using two primer pairs, one targeting telomere repeats (T) and another targeting a known single-copy gene (S)^64^. The ratio between the T and S amplification products (*T*/*S* ratio) is calculated and this correlates with TL; the relative difference in *T*/*S* ratio between samples is proportional to the relative difference in TL. Although it does not require a large amount of starting DNA, it does not give an absolute TL value and there may be substantial variation between cohorts due to different single-copy loci used^61,64^.

Quantification fluorescence in-situ hybridisation (Q-FISH) has several variations that have been used. Q-FISH quantifies the fluorescence intensity after hybridisation with a nucleic acid telomeric repeat, which is then compared against a reference population for a comparative assessment of TL^65^. Flow-FISH is a commonly used variation that can be used for clinical purposes. It combines FISH with flow cytometry, using labelled peptide nucleic acid probes, which hybridise to telomeric repeats in cells and allows measurement of TL in specific cell subpopulations^66^. Q-FISH results are reproducible but it is labour intensive.

Some methods used in research settings include Single-Telomere Length Analysis (STELA), which uses a single-molecule PCR to generate highly accurate TL measurements on individual chromosomes, and Telomere Shortest Length Assay (TeSLA), which measures the shortest telomeres and their longitudinal changes^67,68^. These methods are labour intensive, and low throughput^67,68^. The methods available to measure TL are summarised in Table 3; the selection of the test would also depend on the purpose as large-scale population-based studies would have different requirements from clinical or mechanistic ones.

**Table 3:** Summary of methods to measure telomere length.

Method	Principle	Analyte	Results	Strengths	Limitations
TRF^a^^61,63^	DNA fragments are visualised by Southern blot following digestion, and then compared against DNA ladder.	DNA (0.5–10 µg)	Average TL^k^	▪ Reproducible ▪ Gives direct measurement of TL	▪ Large amount of DNA required. ▪ Subtelomeric polymorphisms impact data. ▪ Low hybridisation efficiency for very short telomeres. ▪ Risk of DNA degradation. ▪ Variation between labs (different restriction enzymes used).
qPCR^b^ mmqPCR^c^ aTLqPCR^d^^61,64^	qPCR: measures ratio between the telomere (T) and single-copy gene (S) amplification products mmqPCR: Telomere DNA and single-copy DNA amplified in the same tube. aTLqPCR: Standard curve from known TL	DNA (<100 ng)	*T*/*S* ratio^l^ (relative quantification)	qPCR: ▪ Small amount of DNA required ▪ High-throughput mmqPCR: ▪ Reduced human error related inaccuracy in qPCR aTLqPCR: ▪ Standard single-copy gene reference	▪ Large variation between different labs (different single-copy loci used). ▪ May not be of use in aneuploidy (single-copy gene duplicated or lost). ▪ Does not detect telomere-free ends ▪ Does not measure individual-specific lengths
STELA^e^ U-STELA^f^ ^61,68^	STELA: Telomeric DNA amplified using subtelomeric- specific primers. U-STELA: DNA digestion followed by specific amplification for short telomeric fragments	Cells (1–1 × 10^5)^	Chromosome-specific TL	Both: ▪ Small amount of material required. ▪ Does not require viable cells STELA: ▪ Detects shortest telomeres on specific chromosomes. U-STELA: ▪ Detects shortest telomeres from every chromosome	Both: ▪ Unable to measure long TL. ▪ Sensitive to DNA input amount. ▪ Labour intensive. ▪ Low throughput. STELA: ▪ Requires highly specific subtelomeric sequences.
TeSLA^g^^67^	Southern blot with hypersensitive digoxigenin-labelled probes	DNA (<1 mcg)	Chromosome-specific TL	▪ Measures very short TL ▪ Detects longitudinal changes in TL	▪ Less suitable for cells with long heterogenous TL. ▪ Labour intensive. ▪ Low throughput.
Q-FISH^h^ (Interphase Q-FISH; Metaphase Q-FISH)^61,65,66^	Telomere fluorescent intensity visualised after hybridisation with (CCCTAA)_3_ probe.	Interphase Q-FISH: Interphase cells (can be done on fixed tissues and cells) Metaphase Q-FISH: Actively dividing cells (15–20 metaphase chromosomes)	Interphase Q-FISH: Average TL measured as relative fluorescence unit Metaphase Q-FISH: Average TL, chromosome-specific TL (both measured as relative fluorescence unit)	Interphase Q-FISH: ▪ Provides telomere length and histological information. ▪ Higher resolution. ▪ Less labour intensive than Metaphase Q-FISH. ▪ High-throughput Q-FISH available. ▪ Does not require mitotically active cells Metaphase Q-FISH: ▪ Suitable for telomeres of various lengths. ▪ Recognises “telomere-free” ends. ▪ Higher accuracy than Interphase Q-FISH.	Both ▪ Relative quantification. ▪ Labour intensive. Interphase Q-FISH: ▪ Unable to detect “telomere-free” ends. Metaphase Q-FISH: ▪ Requires mitotically active cells. ▪ Does not detect telomeres that are very short and do not hybridise with the probe. ▪ Skilled expertise needed for analysis
Flow FISH^61,66^	Combination of flow cytometry with hybridisation of pantelomeric (CCCTAA)_3_ probe to cells in suspension	White blood cells (0.5–2 × 10^6^)	Cell-specific average TL measured as relative fluorescent unit	▪ Cells can be sorted into subpopulations. ▪ Provides 3D telomeric signals within cells. ▪ May be adapted for a higher throughput.	▪ Sensitive to cell types (mostly done on peripheral blood mononuclear cells). ▪ Challenging to process suspension cells. ▪ Non-specific binding of telomeric probe. ▪ Does not detect chromosome-specific individual TL or telomere-free ends. ▪ Labour intensive. ▪ Costly.
PRINS^i61^	Labels telomeric sequences in-situ on metaphase chromosomes/ interphase nuclei	15–20 metaphase chromosomes	Average TL and chromosome-specific TL (both are measured as relative fluorescence unit)	▪ Higher resolution ▪ Measures TL in specific cell types ▪ Can detect individual telomeres, telomere-free ends and average TL when used on metaphase chromosomes.	▪ Labour intensive. ▪ Relative quantification. ▪ Mitotically active cells required for metaphase chromosomes.
HPA^j^^61^	Compares telomeric repeats and Alu repeats	10–3000 ng DNA	Average TL	▪ Quick ▪ Small amount of DNA required	▪ Measures mean telomere length only ▪ Alu repeats in sample can vary

References are in numerical superscript.

^a^Terminal restriction fragment.

^b^Quantitative polymerase chain reaction.

^c^ Monochrome multiplex polymerase chain reaction.

^d^Absolute telomere length quantitative polymerase chain reaction.

^e^Single-telomere length analysis.

^f^Universal single-telomere length analysis.

^g^Telomere shortest length assay.

^h^Quantitative fluorescence in-situ hybridisation.

^i^Primed in-situ subtype of Q-FISH.

^j^Hybridisation protection assay.

^k^Telomere length.

^l^Telomere repeat to single-copy gene ratio.

### Diagnosing TBD

Recognising and diagnosing TBD can be challenging due to the spectrum of presentations (Fig. 4). Heterogeneity within a pedigree exists due to incomplete penetration, anticipation and the influence of TL on disease severity^5–7,17,20^. Environmental insults like smoking may result in genetically predisposed individuals developing IPF earlier in life than nonsmokers, or the use of immunosuppression may result in increased susceptibility to bone marrow failure^5,15,16,52^. In addition, *TERC* and *TERT* mutations may present as isolated AA or IPF and be regarded as sporadic^5–7,54^. Furthermore, not all mutations have been identified and some cases thus remain genetically uncharacterised.

**Fig. 6:**
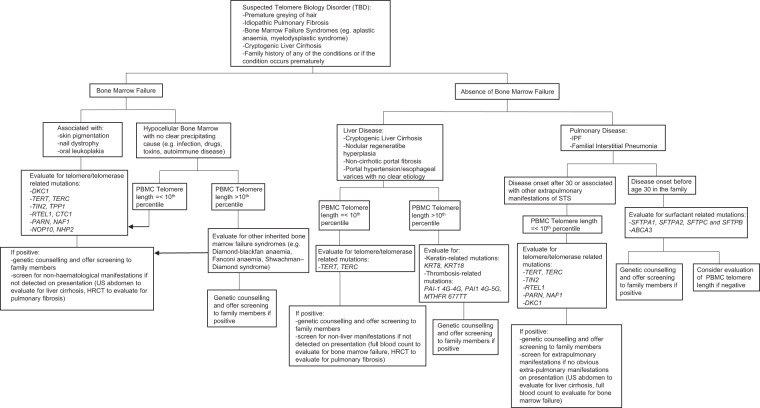
Proposed pathway for evaluation of suspected telomere biology disorders. HRCT, high-resolution computed tomography scan; IPF, idiopathic pulmonary fibrosis; PBMC, peripheral blood mononuclear cell; US, ultrasound.

Diagnostic criteria based on the various systems such as pulmonary, haematological and liver should be defined to help facilitate recognition of TBD. A thorough family history and evaluation of other involved organ systems should be considered and we propose a possible schema to guide clinicians (Fig. 6).

## Management

### Transplantation and immunosuppression

There is no specific treatment for TBD and only transplant is curative. However, immunosuppression is often poorly tolerated and outcomes are worse in TBD. Shorter TL correlate with reduced survival and a faster onset of severe chronic lung allograft dysfunction in pulmonary fibrosis patients who undergo lung transplantation^51^. TBD lung transplant recipients also have a shorter median survival ranging 214 days to 1.9 years compared to non-TBD recipients^69^. Solid organ transplant recipient with TBD are at higher risk of complications such as severe cytopenias, higher infection rates, bone marrow failure, renal complications and calcineurin inhibitor toxicity^69–73^. Furthermore, myeloablative regimens used in bone marrow transplantation for TBD are associated with poorer survival and high rates of pulmonary, liver and endothelial related complications, including unusual complications like veno-occlusive disease^15,74^. Although non-myeloablative fludarabine-based regimens have fewer complications and allow for successful engraftments, TBD recipients may still develop complications in organs that were unaffected prior to transplantation^15,75^.

To date, seven liver transplants in TBD are reported with decompensated liver cirrhosis as the most common indication^60,71–73,76,77^. A combined liver and lung transplant has also been performed for noncirrhotic hepatopulmonary syndrome in end-stage pulmonary fibrosis^71^. Although there is now more experience in performing transplantation in TBD, it remains high risk and poses challenges. Further experience is needed to draw conclusions longer term outcomes of transplant in TBD, particularly with regards to liver and combined organ transplantations.

### Therapeutics

#### Androgen hormonal therapy

Androgens have been shown to improve blood counts, transfusion dependence and liver fibrosis, while also stabilising lung function in TBD patients^78,79^. Although androgens directly increase *TERT* transcription and normalise telomerase activity in mouse models, effects on TL in clinical studies is mixed, with some demonstrating improvement while others have attrition rates similar to placebo group^78,80,81^. Further studies are still required before danazol can be routinely used in TBD and potential adverse effects should be considered. Common complications include transaminitis, muscle cramps, lipid abnormalities and virilisation, while rare complications such as splenic peliosis and rupture have been described with concomitant granulocyte colony-stimulating factor (GSCF) use^78–80^.

#### Non-transplant treatment in IPF

Treatment options are very limited for patients with IPF and currently only the antifibrotics pirfenidone and nintedanib are approved. They retard the rate of forced vital capacity deterioration by 50% a year and pooled analysis suggests mortality benefit, but they are non-curative and do not improve symptoms nor quality of life^82,83^. They are used to treat FIP; however patients have a persistently more aggressive trajectory compared to sporadic IPF and lung transplantation should be explored early in suitable patients^84^.

#### Non-transplant treatment for bone marrow failure

In acquired severe aplastic anaemia where transplant is not suitable, immunosuppression with the use of growth factors like GCSF, erythropoietin and eltrombopag can improve treatment response and accelerate count recovery^85,86^. GCSF does not improve trilineage response or overall survival, and potentially increases the risk of secondary clonal disorders like AML and MDS^85,87^. Concomitant use of GCSF with danazol should be used cautiously or avoided if possible due to the risk of splenic peliosis and rupture^79,80^. Eltrombopag has been shown to restore haematopoeisis even after discontinuation, however further study is needed before this can be used as monotherapy in severe aplastic anaemia^86^.

MDS should be managed according to risk stratification, lower risk patients may be considered for immunosuppression, thrombopoiesis-stimulating agents or DNA-hypomethylation agents such as azacytidine^88^. In higher risk non-transplant candidates, DNA-hypomethylation agents may be used as tolerated^88^. Supportive treatments like blood transfusions alleviate symptoms such as fatigue, and antimicrobial prophylaxis reduces the risk of opportunistic infections^89^.

#### Non-transplant treatment of liver cirrhosis

Advanced liver cirrhosis is often complicated by portal hypertension with sequelae like ascites, oesophageal varices, and HPS^90^. This may lead to fatal gastrointestinal tract bleeding and multi-organ failure. Medications like non-selective beta-blockers to reduce portal venous pressures and endoscopic interventions such as endoscopic band ligation to reduce variceal bleeding risk are often required^90^. Ascites is the most common cause of decompensated liver cirrhosis and a moderate sodium-restrictive diet with diuretics should be prescribed; large volume paracentesis may be required in symptomatic or massive ascites^90^. Hepatotoxic drugs and alcohol should be avoided, and laxatives prescribed to prevent constipation, which may precipitate hepatic encephalopathy. Patients with recurrent hepatic encephalopathy may require rifaximin prophylaxis^90^.

### Potential new therapies and trials

Imetelstat is a 13-mer lipid-conjugated oligonucleotide that competitively inhibits telomerase and has been shown to inhibit cellular proliferation in tumour xenografts and cancer^91^. A pilot study in myelofibrosis demonstrated that 21% achieved partial or complete remission, while another trial in advanced non-small cell lung cancer showed a trend towards improvement in progression-free and overall survival in patients with short telomeres^92,93^. The main adverse effects were severe myelosuppression and transaminitis^92,93^. Further trials are underway to evaluate safety profile and efficacy.

Other investigational treatments include small molecule PAPD5 inhibitors, TA-65 and telomerase gene therapy^94–96^. PAPD5 inhibitors like BCH001 and RG7834, demonstrated restoration of telomerase activity and TL in DC induced pluripotent stem cells and mouse models^94^. TA-65 is a small molecule activator of telomerase that extended TL compared to placebo in healthy subjects^95^. Telomerase gene therapy using adenovirus-associated virus (AAV)9 gene therapy vectors has been shown to lengthen telomeres, improve blood counts and survival in mice with aplastic anaemia^96^. However, further studies are needed to evaluate safety and outcomes before these can be translated to further trials.

### Quality of life

Early palliation should be commenced in TBD patients not suitable for transplant. Common areas of need identified in patients with bone marrow failure, IPF and advanced liver disease include improving quality of life through management of symptoms such as fatigue, dyspnoea and pain, psychological support, advanced care planning, and access to resources like specialised care and hospice services^89,97–99^. The disease burden in patients with single organ disease is high and may compounded in those with multisystem involvement.

### Genetic counselling and testing

TBD is rare and complex with limited treatment options. A certain level of genetic literacy is thus needed for patients to make challenging informed decisions and share information with relatives^100^. Furthermore, inaccurate informal sources may further hinder understanding^100^. Although genetic counselling and patient education initiatives improve knowledge, surveyed patients still answer less than 60% of disease-specific and general genetic knowledge questions correctly when assessed, suggesting patient education has further room for improvement^100^.

In suspected cases of inherited bone marrow failure syndrome, other conditions to consider besides TBD would include Diamond-Blackfan anaemia, Fanconi anaemia and Shwachman–Diamond syndrome^15^. IPF cases are not routinely offered genetic counselling unless they present young and have features suggestive of FIP, due to a low pretest probability^44,47^. Patients with short TL should be offered screening for TBD, while those with a personal or family history of early-onset lung malignancy or disease, should be counselled for surfactant-related mutation screening^45^ (Fig. 6). A thorough assessment should be done prior to offering genetic testing with appropriate counselling on the risks and benefits, as a confirmatory diagnosis has far-reaching implications.

## Outlook

Since DC was first described, there have been significant developments in telomere biology and its implications on senescence and disease pathogenesis. TBD is a syndrome of premature aging and the prototype for degenerative diseases, as telomeres shorten with age. It is likely the most common monogenic premature aging disorder and identifying its syndromic nature has important clinical implications as seemingly sporadic diseases share a common pathways of telomere dysfunction. Its role in more complex pathogenesis such as carcinogenesis, suggests that genotype–phenotype correlations extend beyond what is currently understood. Other mechanisms of telomere dysfunction and mutations will likely be identified with further genotype–phenotype correlations.

Future research would include standardisation of TL measurement methods and benchmarking TL across different cell types and ages. This would allow for comparison of different study results and establishing a gold standard for clinical testing, where TL potentially could have utility as a biomarker of disease predisposition, prognosis and treatment response.

Establishing clinical and genetic criteria for TBD diagnosis would be invaluable for risk stratification and genetic counselling. Increased awareness and education of medical practitioners can help early recognition and facilitate diagnosis. Multidisciplinary genetics clinics with genetic counselling and support services would also help improve care. Early advanced care planning should be initiated with open discussions to explore patients’ ideas, concerns, and expectations so that appropriate supportive care is received in a timely manner.

Regarding therapeutics, androgen treatment and novel therapies targeting telomere homoeostasis pathways are areas for development. These will require further trials before they are suitable for clinical use. If successful, given the involvement of telomere dysfunction in various disease pathways, such treatments could potentially be used to treat other diseases beyond TBD that are currently incurable.

Although various aspects of care discussed in this review are regarded as standard of care in TBD, in many parts of the world, these services and enrolment into trials may not be available or easily accessible to patients. Greater awareness of TBD is needed to improve overall care of patients with this disease.

### Reporting summary

Further information on research design is available in the Nature Research Reporting Summary linked to this article.

## Supplementary information

Reporting Summary
